# Graph Neural Network and Context-Aware Based User Behavior Prediction and Recommendation System Research

**DOI:** 10.1155/2020/8812370

**Published:** 2020-11-30

**Authors:** Qian Gao, Pengcheng Ma

**Affiliations:** School of Computer Science and Technology, Qilu University of Technology (Shandong Academy of Sciences), Jinan, Shandong 250353, China

## Abstract

Due to the influence of context information on user behavior, context-aware recommendation system (CARS) has attracted extensive attention in recent years. The most advanced context-aware recommendation system maps the original multi-field features into a shared hidden space and then simply connects it to a deep neural network (DNN) or other specially designed networks. However, for different areas, the ability of modeling complex interactions in a sufficiently flexible and explicit way is limited by the simple unstructured combination of feature fields. Therefore, it is hard to get the accurate results of the user behavior prediction. In this paper, a graph structure is used to establish the interaction between context and users/items. Through modeling user behavior, we can explore user preferences in different context environments, so as to make personalized recommendations for users. In particular, we construct a context-user and context-item interactions graph separately. In the interactions graph, each node is composed of a user feature field, an item feature field, and a feature field of different contexts. Different feature fields can interact through edges. Therefore, the task of modeling feature interaction can be transformed into modeling the node interaction on the corresponding graph. To this end, an innovative model called context-aware graph neural network (CA-GNN) model is designed. Furthermore, in order to obtain more accurate and efficient recommendation results, first, we innovatively use the attention mechanism to improve the interpretability of CA-GNN; second, we innovatively use the degree of physical fatigue features which has never been used in traditional CARS as critical contextual feature information into our CA-GNN. We simulated the Food and Yelp datasets. The experimental results show that CA-GNN is better than other methods in terms of root mean square error (RMSE) and mean absolute error (MAE).

## 1. Introduction

**Figure 1:**
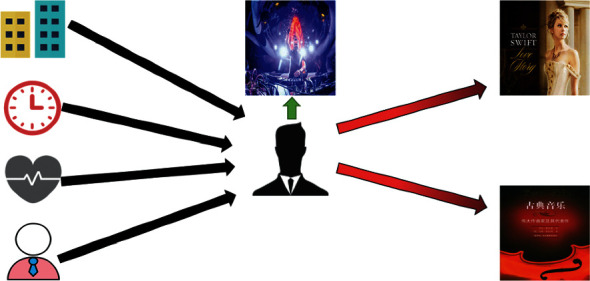
The influence of context on user behavior.

It is fundamental to establish a model to capture the user's interest and interaction with the items in the recommendation system. In addition, the additional context information [1] such as the degree of physical fatigue and other contextual information on the interaction with the users/items also plays a critical role in the accuracy for all types of recommendation systems. As illustrated in Figure 1, a user usually likes to listen to some electronic passion songs, but when he is at home with his wife at night and feels relaxed, he prefers to listen to warm love songs. And when he is in the company during the day and is nervous, he prefers to listen to some soothing classical piano music. This example shows that the user's music preference is easy to be affected by the specific contextual environment features (the contextual environment features in the above example include time, place, companionship, and degree of physical fatigue). Therefore, it is necessary to consider the context in the recommendation system; context has a great influence on users' behavior. In particular, relaxation and nervousness indicate different degrees of physical fatigue. In the figure, the black arrows represent different contextual information that can affect the user's interests. The green arrow indicates the user's personal interest, and the red arrow represents the change of the user's interest as the context changes.

The importance of context has been widely recognized in the field of recommendation systems. Most of the earlier studies [2] are low-order feature interactions modeling methods. They can only obtain the linear relationship between features by expanding the latent factor model to integrate contextual information; the context is usually regarded as the additional dimensions similar to users and items, so as to calculate the relevance between the context and users/items. The problem for this kind of approach is that it is difficult to explain the complex relationship between the context and the user/item. To solve this problem, Shi et al. [3] proposed a new latent factor model based on the context-aware representation of users and items. These representations are learned by adding additional layers of potential space for users/items in a given context. Liu et al. [4] and Wu et al. [5] proposed the context operating tensor (COT) model, which represents context as a latent vector. However, the above methods rely on linear operations (matrix factorization [6]) to model the observed data and can only obtain simple low-order feature interaction relationships between features. They are not suitable for the actual situation with much more complicated interaction relationship.

Mei et al. [7] proposed a method to reformulate CARS tasks by designing different objects (users, items, and contexts) and interactions (context-user interaction, context-item interaction, and user-item interactions), which makes a clear difference between different types of interactions. Then, a new neural model called the attention interactive network (AIN) was established. AIN explores methods of using deep neural networks to model the interaction of context on the user and the item representation. However, AIN only simply maps the original multi-field features into a shared hidden space and then simply connects them to the multilayer perceptron (MLP) to learn the high-order feature interactions between users/items and the context. Therefore, the simple unstructured combination of functional fields will inevitably limit the ability to model complex interactions between different fields in a flexible and explicit manner. Therefore we cannot obtain the accurate high-order feature interactions relationship.

Traditional CARS only use time and place as the context environment [8, 9], but the actual context environment is more complex. All features that have an interactive impact on users and items can be used as context features. Therefore, in addition to time and place, other context, which can affect the user's preference, should also be considered. Previous recommendation systems have not been studied in this regard, which has led to the failure to detect short-term real-time preferences of users in time. For example, users may take a break from the work and temporarily browse entertainment news to relax, and users also may order some drinks or food to help them relieve fatigue. Therefore, short-term real-time preferences are different from the long-term preferences during the work due to the context. If the research on the content of the degree of physical fatigue feature can be added to the context-aware recommendation system research, the user's short-term real-time preferences can be found in time and the items with high user satisfaction can be recommended to users accordingly.

In this paper, we innovatively take the degree of physical fatigue as a contextual feature, because the degree of physical fatigue often affects the user's subsequent behavior.

In view of the problem that the previous recommendation system could not model the complex interaction between different features in a flexible and display way, as well as the problem of neglecting the degree of physical fatigue features, in this paper, we innovatively use graph neural network to model and add the degree of physical fatigue features. The graph neural network can not only discover users' short-term preferences in time, but also model the complex interaction between different features in a flexible and display way, which will greatly improve the accuracy of the recommendation system. Furthermore, we innovatively take the degree of physical fatigue as a contextual feature, which often affects the user's subsequent behavior.

Our contributions can be summarized in four aspects:

1. This paper innovatively establishes a CA-GNN to model the context-user and context-item interactions on graph structure features in a more flexible and explicit way; the CA-GNN improves the prediction accuracy of the recommendation system greatly.
2. We use a single-layer perceptron model to calculate the attention edge weight, through which we can find the influence degree between different nodes. And we innovatively use the attention mechanism to improve the interpretability of CA-GNN.
3. This paper innovatively takes the degree of physical fatigue as a key contextual feature, which makes up for the shortcomings of traditional CARS that only consider time and place in the past.
4. The simulation based on the two real rating datasets shows that our proposed method can surpass the existing methods.

## 2. Related Work

### 2.1. Traditional Context-Aware Recommendation System

Adomavicius and Tuzhilin [10] have proved that, in context-aware recommendation research, the incorporating contextual information can improve the accuracy of recommendation. According to the contextual information used in the recommendation process at different stages, the context-aware recommendation algorithm is divided into three paradigms: contextual prefiltering, contextual postfiltering, and contextual modeling. The contextual prefiltering paradigm [11] uses context information for data selection and data construction, that is, using the current context information to filter the original dataset, constructs a dataset related to the current context information, and finally makes recommendations. The contextual postfiltering paradigm [12] uses context information to filter or reorder the recommendation results generated by traditional two-dimensional recommendation techniques. Although context prefiltering and postcontext filtering may work in practice, they need to be supervised and fine-tuned in all steps of the recommendation. Furthermore, in the process of dimensionality reduction, the certain correlation between different context information pieces is ignored.

Different from the previous two paradigms, the contextual modeling paradigm directly considers context information in the modeling process and has become the most popular method now. According to previous research, the current contextual modeling paradigm can be roughly divided into two categories: one is the contextual modeling method of low-order feature interactions and the other is the contextual modeling method of high-order feature interactions. Most of the low-order feature interaction models are based on matrix factorization (MF) [13–15] and factorization machine (FM) [16, 17]; however, these methods regard the context as some feature objects of the user or item. Therefore, the correlation between the context and the user/item is not intuitive and unexplainable. In response to the above problems, Liu et al. and Wu et al. proposed the COT [4, 5], which represents the common semantic effect of context on users and items as a context-operating tensor and represents the context as latent vector representation. Then, the model generates the context operation matrix through the context operation tensor and the context latent vector representation and then performs the inner product operation on the context operation matrix and the user latent vector representation (or item latent vector representation). Finally, the obtained inner product operation results are used to model and generate a context-aware user representation (or context-aware item representation). COT is a high-order feature interactions model, but the interaction between the context and the user (or context and item) is modeled in a linear manner (for example, matrix decomposition), which makes it difficult to model the role and impact of nonlinearity between context and users/items in actual situations.

Overall, the study of high-order feature interactions models is later than the study of low-order feature interaction models. Most of the recent high-order feature interaction models use the deep learning model to learn the high-order feature interaction, which can learn the high-order and complex nonlinear relationship between different features. We will introduce this kind of high-order feature interaction model in detail in the next section.

### 2.2. Deep Learning Methods

In recent years, deep learning methods have received great attention and have been applied in various fields. Using deep learning methods can learn high-order and complex nonlinear relationships between different features; therefore, the context-aware recommendation system has also begun to try to use deep learning methods.

The FM based deep learning method, which is called neural factorization machine (NFM) model, was proposed by He and Chua for sparse data prediction [18] to model high-order nonlinear feature interactions. In 2018, the research group of Lei Mei in Shandong University redefined context-aware recommendation task under the inspiration of physical reasoning task [19]. They proposed an innovative neural network model, which first captures the interaction between the context and the user (or item) and obtains the effect of each context on the user (or item) and then learns the importance of each context with the help of attention mechanism [20]. Finally, the overall impact of the current context on the user (or item) is obtained through a synthesis operation, thereby modeling the changes in user's interest or item attributes according to the influence of context. However, this deep learning based model follows a common paradigm: first map the original multi-field features into a shared hidden space as input data, and then simply connect it to a DNN or other specially designed networks to learn high-order feature interactions. However, the simple unstructured combination of feature fields will inevitably limit the ability to model complex interactions between different fields in a sufficiently flexible and explicit way, which leads to the fact that AIN cannot find the real influence relationship between features. Xiao et al. also introduced an FM model based attention mechanism [21] and proposed an attentional factorization machine (AFM) model [22]. AFM model uses attention mechanism to enhance the interpretability of recommendation system, but it can only build low-order linear feature interaction, but not high-order nonlinear feature interaction. Although NFM and AFM are not specific context-aware recommendation models, both can be applied to the CARS by specifying input data.

In recent years, the deep learning based graph neural network (GNN) [23] becomes a typical graph analysis method. Nodes in GNN interact with neighbor nodes by aggregating information from neighbors and updating their hidden states. Due to the convincing performance and high interpretability of GNN, it has been used in recommendation systems [24]. GNN is essentially suitable for modeling node interactions on graph structural features; it can model the complex interaction between different fields in a sufficiently flexible and explicit way. In the article “Gated Graph Sequence Neural Networks” (GGNN) [25] published in 2016, Li et al. mentioned that node information can be obtained by summation of neighbor nodes; furthermore, the GRU is used as an updater in GGNN. Li et al. established Fi-GNN model [26] on the basis of GGNN, which models feature interaction based on graph structure features. Fi-GNN uses a graph structure to intuitively represent the characteristics of multiple fields, in which each node corresponds to a feature field, and different fields can interact through edges. Therefore, the task of modeling feature interaction can be transformed into modeling the interaction of nodes on the corresponding graph. The CA-GNN model in this paper is based on Fi-GNN model. In the context-aware recommendation system, the interaction between user (or item) and context is modeled based on the attributes of nodes and edges in the graph structure, so as to achieve the purpose of refining the user (item) representation. This interaction is realized by the edges between nodes in the graph. In fact, the information of neighbor nodes is aggregated by edges, and then the hidden state of itself is constantly updated through GRU.

### 2.3. Physical Fatigue

Fatigue is commonly referred to as a feeling of tiredness, which means a lack of energy and vitality in the body. Fatigue is different from depression. It is closely related to the impairment of the body or cognitive function. When people are fatigued, they often feel sleepy and feel difficulty in concentrating on anything [27]. In the fields of transportation, learning, and shopping, physical fatigue can have a huge negative impact on users, sometimes even threatening life. Under normal circumstances, when users feel tired, they will choose to rest or buy some food and drinks that quickly relieve fatigue in a short period. In the shopping field, the user's physical fatigue degree feature can directly affect the user's item preferences. Therefore, effectively identifying the user's degree of physical fatigue feature is very meaningful for the research of recommendation systems.

Ma and Gao [28] of Qilu University of Technology used wavelet transform to extract the features of EEG data and then construct a depth factorization machine model (FM + LSTM) which is composed of factorization machine (FM) and long short-term memory (LSTM) [29] in parallel to predict the user's eye state (open or closed eyes). With the help of this study, people can determine the user's degree of physical fatigue by detecting the user's eye state for a long time. Zeng et al. from Changchun University of Science and Technology [30] designed and implemented an electroencephalogram based fatigue detection experiment and collected the electroencephalogram of the subject and recorded the eye image data of the subject. Using wavelet transform to extract features of EEG signals, analyze the changes in the degree of physical fatigue of the subjects during the experiment. At the same time, process the eye image data of the subject, count the number of blinks of the subject, and analyze the changing trend. The conclusion that blink rate increases with the increase of fatigue was verified objectively based on the analysis of the correlation between EEG data and blink rate. The above work provides a theoretical basis for diagnosing the user's physical fatigue through EEG data and shows that the degree of physical fatigue is very important in the current research. This is also an important reason for the study of the impact of body fatigue as a context on user behavior.

### 2.4. The Research Content of This Paper

From the above analysis, we can see that the low-order feature interaction models are difficult to learn complex feature interaction relationships, and the high-order feature interaction model lacks the ability to model the complex interaction between different fields in a flexible and explicit way. This paper attempts to establish the interaction between context and users/items through the graph, and we innovatively use the degree of physical fatigue features that are not used in traditional CARS as key contextual feature information. In particular, to effectively distinguish the different effects of given context information on users and items, we construct a context-user and context-item interactions graph. In the interactions graph, each node represents a feature field of the user/item and different context environment; different field features can interact through edges. Therefore, the task of modeling feature interactions can be transformed into modeling the node interactions on the corresponding graph.

First, we obtain a degree of physical fatigue as a special context environment feature based on the sweetness of the food in the dataset. Second, we designed a new model called CA-GNN to model the interaction between user/item feature fields and context environment feature fields in a flexible and explicit way by taking advantage of the power of graphs. It can obtain higher accuracy and higher efficiency recommendation results than the above mentioned approaches. We also use the attention mechanism to improve the interpretability of the model. Simulation shows that the proposed CA-GNN with the degree of physical fatigue feature has greatly improved the accuracy of the recommendation system.

## 3. The Proposed Context-Aware Graph Neural Network (CA-GNN) Model

First of all, we introduce the way of data acquisition, and the mathematical description. On this basis, we further introduce how to use CA-GNN to construct the complex interaction between context field features and user/item feature fields. Each part of this section will be described in detail below.

### 3.1. Data Acquisition

#### 3.1.1. Information Obtained by Physical Devices

Context-aware information obtains user device information and user location information through sensor network and adopts traditional broadcast push method. It mainly includes RF identification, temperature and humidity sensor, gas sensor, and other physical sensing devices. The context-aware system is mainly arranged in a relatively closed environment, which has specific requirements for indoor environment, such as in rooms, restaurants, conference halls, offices, schools, hospitals, and other specific places, so the amount of data transmitted is determined by the activity transaction volume of these places. For places such as offices, the amount of data is relatively small due to the small amount of personnel activities, while for places such as hospitals, the amount of data is large due to the large amount of personnel activities.

#### 3.1.2. Information Obtained by Application Software

Through the log file and other information management system software, the user related context information is obtained from the interactive user interface. In addition, the protocol package created by the mobile terminal is collected through WLAN network; the login data of users on social network (or application), e-commerce website (or application), e-book reading website (or application), e-mail service network and registration, and preference information provided and authorized by users are analyzed. Then, the basic information about users and the main information of users preference is obtained.

The Food and Yelp datasets used in this paper are obtained by related individuals or organizations through the above two ways.

### 3.2. Problem Formulation

Suppose that the training dataset is composed of user *u* feature domain, item *v* feature field, context *c*1 feature field, context *c*2 feature domain, context *c*3 feature field, and related tag *R* to represent the user's scoring behavior. Therefore, the training set contains five feature domains (*f* = 5). The user rating task is to predict the user's estimated score $\hat{R}$ by inputting the context-user feature field (including 4 feature domains) and context-item feature field (including 4 feature domains). The key of the task is to model the complex interaction between context-user feature domain and context-item feature domain, so as to achieve the purpose of refining user (item) representation, and finally predict the user's estimated score $\hat{R}$ through user item interaction.

In this paper, we study the problem of rating prediction in recommendation system; the actual user rating *R* ∈ (1,2,3,4,5). After a lot of verification [7], the actual recommendation system does not regard the score prediction as a multi-classification task. The purpose of score prediction is to get the difference between the score of recommendation prediction in all test sets and the score of actual users. The common evaluation indexes of this kind of task are root mean square error (RMSE) and mean absolute error (MAE), so this is a regression prediction problem.

### 3.3. Overview

**Figure 2:**
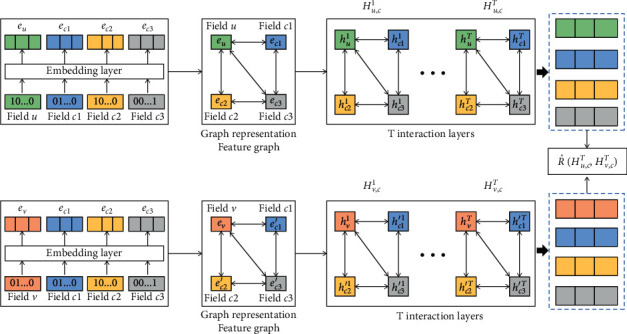
Flowchart of establishing modeling in this paper.

Figure 2 is an overview of our proposed method. Firstly, the context user domain feature vectors and context item domain feature vectors are mapped into sparse single hot embedding vectors and then embedded into dense field embedding vectors through embedding layer. In this way, we construct context-user and context-item feature graphs, in which each node corresponds to a feature field, and different feature fields can interact through edges. Therefore, the task of modeling interaction can be transformed into modeling node interaction on feature graph. Then, the feature graph is added to the CA-GNN to model the node interaction. Finally, the user estimated score $\hat{R}$ is predicted through user-item interaction (the simple interaction function of inner product) on the output layer of CA-GNN.

In this paper, CA-GNN is established by using graph neural network. The embedded input layer in CA-GNN is to represent each field in context-user dataset and context-item dataset as one-hot coded vector and then embed one-hot coded vector into a dense vector to obtain low-dimensional context user (item) feature vectors. The output layer of CA-GNN is implemented by user-item interaction, which actually uses a point product function. Context information in recommendation system refers to the time, place, and mood of users visiting the recommendation system. And the context information in different datasets is different. The most commonly used information is time and place information, but not all datasets contain these two types of information. Specific to a certain dataset, we must select reasonable information as the context. For example, the Food dataset used in this paper does not contain time and place. We choose virtuality, hunger, and fatigue as the context because they can have a certain impact on users' interests and item attributes. In addition, in the Yelp dataset, we select year, month, day (a day of the week), and city as contexts, which are related to time (year, month, day) and place (city). The interaction refers to the learning of the cross combination of two or more features. The feature interaction in this paper is realized by using the edge between nodes in graph structure.

### 3.4. Embedding Layer

The feature fields in context-user and context-item are usually very sparse and high dimensional, so we need to represent them as field embedding vectors. First, we represent each field as a one-hot encoding vector [31] and then embed it into a dense vector. Let us consider an example. A user usually goes to the restaurant by himself and likes to eat some fried food such as French fries. He will give a high score every time he buys this kind of food. At work, the user will be very tired and nervous. When the user and his colleagues go to the restaurant for lunch, they will eat some set meal containing rice. For this kind of affordable food, the user will give a high score, but if the price of the selected food is high and the portion size is small, the user will give a very low score. After work, the body is more relaxed. When the user goes to the restaurant with his wife in the evening, they will buy some delicate and delicious food. Each time they choose this kind of food, they will give a high score, but if the selected food is not good-looking and tastes bad, they will give a very low score. Through a one-hot encoding is converted into a high-dimensional sparse feature, as shown below:

(1) $$\begin{array}{c} \left(\begin{array}{cccccc} \underset{\text{User}}{\left[1,0,\ldots ,0\right]}, & \underset{\text{Deliciousfood}}{\left[0,1,\ldots ,0\right]}, & \underset{\text{Evening}}{\left[0,1,\ldots 0\right]}, & \underset{\text{Wife}}{\left[0,1,\ldots ,0\right]}, & \underset{\text{Home}}{\left[0,1,\ldots ,0\right]}, & \underset{\text{Notired}}{\left[0,1,\ldots ,0\right]} \end{array}\right). \end{array}$$

To obtain the low-dimensional data, we input the one-hot encoding vector into the embedding layer. The context-user feature embedding vector *E*_*u*,*c*_ and context-item feature embedding vector *E*_*v*,*c*_ can be obtained, as shown below:

(2) $$\begin{array}{c} E_{u,c}=\left[e_{u},e_{c1},e_{c2},\ldots ,e_{ck}\right],\\ E_{v,c}=\left[e_{v},e_{c1}^{\prime },e_{c2}^{\prime },\ldots ,e_{ck}^{\prime }\right]. \end{array}$$

Among them, *e*_*u*_ ∈ *R*^*D*^, *e*_*v*_ ∈ *R*^*D*^, *e*_*ck*_ ∈ *R*^*D*^, and *e*_*ck*_′ ∈ *R*^*D*^ represent the embedding vectors of the fields *u*, *v*, and *c*_*k*_ (including various context feature fields), and *D* represents the dimension of the field embedding vector.

The experimental dataset contains a large number of user consumption score records, so there are many user categories. For example, there are 100 consumer rating records. First of all, the 100 users are coded by one-hot. Then, the first user is (1, 0, 0,…, 0), the second user is (0, 1, 0,…, 0), and the 100th user is (0, 0,…, 0, 1). In other words, the dimension of the user vector encoded by one-hot is 100 dimensions (*D* = 100). After one-hot coding, the dimension of user vector is very high, which leads to the problem of data sparsity. In order to reduce the dimension and get dense embedding vector, we embed it. After embedding, the user vector is no longer as high as 100 dimensions. For example, the dimension after embedding is set to 16 (*D* = 16). Then, after embedding, the dimension of user vector is 16, and the 16-dimensional user embedding vector is randomly initialized and then learned through CA-GNN network training in this paper, so the embedding vector of each user is different. Each item and other contexts embed vectors in the same way as each user.

### 3.5. Constructed Feature Graph

We represent the relationship between each node as a graph structure and then input it into the designed model to learn the interactive relationship of learning features. We constructed a training dataset based context-user/item interactions graph *G*=(*N*, Ε) based on the training dataset. Each node *n*_*i*_ ∈ *N* (representing a feature field *i*) in the graph makes |*N*|=*f* − 1, where ℰ is the edge of two nodes *n*_*i*_ and *n*_*j*_. Because every two nodes should be round-way interactive, this is a weighted, fully connected graph, and the edge weights reflect the interaction between different feature domains.

### 3.6. Context-Aware Graph Neural Network

Our CA-GNN method consists of two steps. The first step is to learn the initial node state. The second step is to model the node interaction and update the node state.

**Figure 3:**
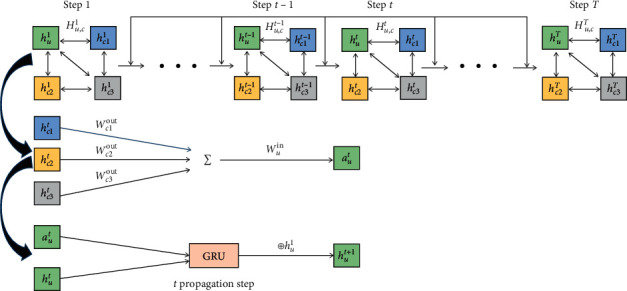
The framework of CA-GNN.

In Figure 3, the nodes interact with neighbors, and their state is updated circularly. Figure 3 only shows the way of modeling context-user feature interactions, and the way of modeling context-item feature interactions is the same. In each interaction step, each node first aggregates the transformed state information from its neighbors and then updates its state based on the aggregated information and history through GRU and the remaining connections. The CA-GNN framework refers to the model framework of Fi-GNN [26], but CA-GNN is a specific implementation method in the field of context-aware recommendation system. There are obvious differences between CA-GNN and Fi-GNN in application fields and implementation details.

#### 3.6.1. Initial Node State

In CA-GNN, each node is associated with a hidden state vector. The state set *H*_*u*,*c*_^*t*^ of each node in the context user interaction diagram and the state set *H*_*v*,*c*_^*t*^ of each node in the context object interaction diagram are shown below:

(3) $$\begin{array}{c} H_{u,c}^{t}=\left[h_{u}^{t},h_{c1}^{t},h_{c2}^{t},\ldots .h_{c_{k}}^{t}\right],\\ H_{v,c}^{t}=\left[h_{v}^{t},h_{c1}^{\prime t},h_{c2}^{\prime t},\ldots .h_{ck}^{\prime t}\right]. \end{array}$$

Among them, *t* represents the steps of interaction. As shown in Figure 3, nodes interact in a circular fashion and update their state. In each interaction step, the node and its neighbors aggregate the transformed state information and then update the node state according to the aggregated information and history through GRU [32] and the remaining connections.

Define the initial status of the start node. The initial node state is the node state of the input feature graph, that is, the field embedding vector. The embedded vector of the field is used as the initial state vector of the corresponding node, which can be formalized as *H*_*u*,*c*_^1^ and *H*_*v*,*c*_^1^, as shown below:

(4) $$\begin{array}{c} H_{u,c}^{1}=\left[e_{u}^{1},e_{c1}^{1},e_{c2}^{1},\ldots .e_{ck}^{1}\right], \end{array}$$

where *h*_*u*_^1^=*e*_*u*_^1^, *h*_*v*_^1^=*e*_*v*_^1^, *h*_*c*1_^1^=*e*_*c*1_^1^, *h*_*c*1_^′1^=*e*_*c*1_^′1^, *h*_*c*2_^1^=*e*_*c*2_^1^, *h*′_*c*2_^1^=*e*′_*c*2_^1^, *h*_*ck*_^1^=*e*_*ck*_^1^, *h*_*ck*_^′1^=*e*_*ck*_^′1^, so *h* ∈ *R*^*D*^, *H* ∈ *R*^(*k*+1)×*D*^.

#### 3.6.2. Modeling Node Interaction

In the traditional GGNN [25], in the propagation step *t*, the sum of neighbor states received by the node is *a*_*i*_^*t*^, as shown in

(5) $$\begin{array}{c} a_{i}^{t}=\sum_{n_{j}⟶n_{i}\in ɛ}A\left[n_{j},n_{i}\right]W_{p}h_{j}^{t-1}+b_{p}. \end{array}$$

Among them, *W*_*p*_ and *b*_*p*_ are the weights and deviations of the shared linear transformation on all sides, and *A*[*n*_*j*_, *n*_*i*_] is the adjacency matrix.

Calculate the attention edge weight. There is an interaction between two nodes in a completely connected graph. In order to infer the importance of the interaction between different nodes, we propose an attention mechanism to learn the edge weights between nodes. Specifically, the weight of node *n*_*i*_ to the edge of node *n*_*j*_ is calculated according to their initial node state. We use single layer perceptron [33] to calculate the attention score, as shown in the following equation:

(6) $$\begin{array}{c} a\left(n_{j},n_{i}\right)=\sigma \left(W_{1}e_{j}+W_{2}e_{i}+b_{1}\right). \end{array}$$

In calculating the attention score *a* of each node on *n*_*i*_, we normalize the above scores by a softmax [34] function to get the final attention weight from *n*_*j*_ to *n*_*i*_, as shown in the following equation:

(7) $$\begin{array}{c} w\left(n_{j},n_{i}\right)=\frac{\exp\left(a\left(n_{j},n_{i}\right)\right)}{\sum_{k}\exp\left(a\left(n_{k},n_{i}\right)\right)}. \end{array}$$

Therefore, we can get the adjacency matrix *A*[*n*_*j*_, *n*_*i*_], as shown in equation (8):

(8) $$\begin{array}{c} \text{A}\left[n_{j},n_{i}\right]=\left\{\begin{array}{cc} w\left(n_{j},n_{i}\right), & i\neq j,\\ 0, & \text{others}. \end{array}\;\right. \end{array}$$

**Figure 4:**
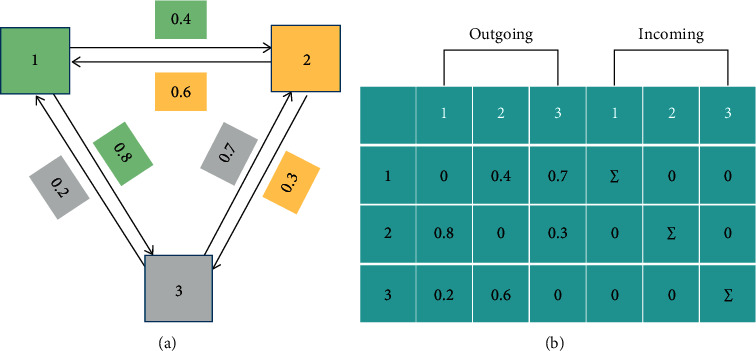
(a) Schematic diagram of edge conversion between nodes. (b) Σ is the sum of the input values of node *n*_*i*_.

In the traditional GGNN, the same *W*_*p*_ and *b*_*p*_ are used to model the interaction between different nodes. GGNN only considers the one-way propagation between two nodes. Still, in the actual graphics, the influence between two points is often mutual, so we must consider the round-way interaction between two nodes. Therefore, we add an output matrix *W*_out_ and an input matrix *W*_in_ [26, 35] to represent the bidirectional interaction between each node *n*_*i*_ and different nodes in CA-GNN.  Figure 4 shows the edge transformation between nodes. The transformation function of the edge *n*_*i*_⟶*n*_*j*_ from node *n*_*i*_ to node *n*_*j*_ is shown in:

(9) $$\begin{array}{c} W_{p}^{n_{j}⟶n_{i}}=W_{\text{out}}^{j}W_{\text{in}}^{i}. \end{array}$$

Therefore, equation (6) can be rewritten as shown in the following equation:

(10) $$\begin{array}{c} a_{i}^{t}=\underset{n_{i}⟶n_{i}\in ɛ}{\sum }A\left[n_{j},n_{i}\right]W_{\text{out}}^{j}W_{\text{in}}^{i}h_{j}^{t-1}+b_{p}. \end{array}$$

After receiving status information *a*_*i*_^*t*^, the status of the node *n*_*i*_ is updated as shown in the following equations:

(11) $$\begin{array}{c} z_{i}^{t}=\sigma \left(W_{z}a_{i}^{t}+U_{z}h_{i}^{t-1}+b_{z}\right), \end{array}$$

(12) $$\begin{array}{c} r_{i}^{t}=\sigma \left(W_{r}a_{i}^{t}+U_{r}h_{i}^{t-1}+b_{r}\right), \end{array}$$

(13) $$\begin{array}{c} {\tilde{h}}_{i}^{t}=\tanh\left(W_{h}a_{i}^{t}+U_{h}\left(r_{i}^{t}⊙h_{i}^{t-1}\right)+b_{h}\right), \end{array}$$

(14) $$\begin{array}{c} h_{i}^{t}={\tilde{h}}_{i}^{t}⊙z_{i}^{t}+h_{i}^{t-1}⊙\left(1-z_{i}^{t}\right). \end{array}$$

Among them, *W*_*z*_, *W*_*r*_, *W*_*h*_, *b*_*z*_, *b*_*r*_, and *b*_*h*_ are the weights and deviations of the update function, similar to gated recursive unit (GRU) [32]. *z*_*i*_^*t*^ and *r*_*i*_^*t*^ are the update gate vector and the reset gate vector, respectively.

The status is updated via remaining connections [36]. Therefore, equation (14) can be rewritten, as shown in the following equation:

(15) $$\begin{array}{c} h_{i}^{t}=\text{GRU}\left(h_{i}^{t-1},a_{i}^{t}\right)+h_{i}^{1}. \end{array}$$

### 3.7. Output Layer

Propagation after step *T*: the hidden state of context-user interaction graph and context-item interaction graph can be obtained, as shown in the following equations:

(16) $$\begin{array}{c} H_{u,c}^{T}=\left[h_{u}^{T},h_{c1}^{T},h_{c2}^{T},\ldots ,h_{ck}^{T}\right], \end{array}$$

(17) $$\begin{array}{c} H_{v,c}^{T}=\left[h_{v}^{T},h_{c1}^{\prime T},h_{c2}^{\prime T},\ldots ,h_{ck}^{\prime T}\right]. \end{array}$$

In the output layer, we add attention mechanism. This is mainly aimed at the following two problems: (1) it is unable to obtain the relationship between the nodes; (2) it is unable to distinguish the impact of different nodes on the prediction results. Because attention mechanism can greatly shorten the distance between features, it can effectively use these features to capture key information. In addition, attention mechanism can be used to measure the impact of each node on the overall prediction, so as to predict the user score more effectively. In the Food dataset, there are user nodes, item nodes, and context nodes (including hunger and virtuality). Different nodes are related. At the same time, in the score prediction problem, each node has different influence. For example, if a user has a partiality for fried chicken, then the user node plays a key role in scoring. Besides, Sprite is the most popular carbonated beverage; many people like to drink it. Therefore, the key to score is the item node. The same is true for nodes in Yelp dataset. Consequently, we use attention mechanism in the output layer to solve those two problems.

Each field node interacts with other features and finally captures the global state information. Here, we use an attention mechanism to measure the impact of each node on the overall prediction. Specifically, the weight of each node can be obtained through the attention mechanism layer, which is similar to the method used to calculate the attention side weight. Firstly, the interaction function between different nodes is obtained by using multi-layer perceptron, then the weight of each node is obtained by using softmax function, and finally the representation value of each node is updated, as shown in the equations:

(18) $$\begin{array}{c} h_{i}^{T\prime }=\frac{\exp\left(\beta \left(n_{j},n_{i}\right)\right)}{\sum_{k}\exp\left(\beta \left(n_{k},n_{i}\right)\right)}\cdot h_{i}^{T}. \end{array}$$

(19) $$\begin{array}{c} H_{u}^{T}=\left[h_{u}^{T^{\prime }},h_{c1}^{T^{\prime }},h_{c2}^{T^{\prime }},\ldots ,h_{ck}^{T^{\prime }}\right]. \end{array}$$

The main concern of this paper is to learn the interaction matrix between context, user, and project and get the hidden state vector of user and item affected by context. Therefore, the simple interaction function of inner product is used in the output layer of the CA-GNN model. The output function is shown in the following equation:

(20) $$\begin{array}{c} \hat{R}={\left(H_{u}^{T}\right)}^{T}\cdot H_{v}^{T}\;. \end{array}$$

### 3.8. Loss Function

Our loss function is defined as shown in equation (21):

(21) $$\begin{array}{c} \text{L}=\sum_{\left(u,v,c\right)\in R_{\text{train}}}{\left(R_{u,v,c}-{\hat{R}}_{u,v,c}\right)}^{2}. \end{array}$$

Among them, *R*_train_ is the training set. When the loss function *L* obtains the minimum value, we will get the optimal training parameters.

## 4. Experiment and Simulation

In this section, we conducted a lot of experiments to verify the following questions:

1. Does our proposed CA-GNN model perform better than the other advanced models?
2. How does the context environment feature affect user behavior and item interaction, and what impact does it have on the prediction results, through modeling context-user feature interaction and modeling context-item feature interaction?
3. What kind of influence does the different context environment features have on the final prediction result, and does the degree of physical fatigue feature have a positive influence on the prediction result?
4. How does the use of attention mechanisms in the output layer affect the prediction results?

Next, we will introduce some basic experimental devices and then answer these questions.

### 4.1. Experimental Dataset

This paper evaluates our proposed CA-GNN model on the following two rating datasets.  Table 1 summarizes the data features of these two rating datasets.

#### 4.1.1. Food

We use the Food dataset [37], which contains 6360 5-level ratings of 20 items by 212 users, and each rating is related to 2 contextual features. One contextual feature describes whether the user rate is virtual or real (2 values: real and virtual), and the second contextual feature describes how hungry the user is (3 values: hungry, normal, and full). The dataset is used to test the rating task.

#### 4.1.2. Yelp

The raw Yelp data is very large but very sparse. The Yelp dataset is downloaded from the website https://www.yelp.com/dataset/challenge and sorted out. The Yelp dataset has large-scale context features. We select a data subset that contains 96143 users, 49482 items, and 2283913 interactions. In the Yelp dataset, we selected the context features including year, month, day of the week, and city.

### 4.2. Model Evaluation Method

In evaluating the CA-GNN score prediction model proposed in this paper, we used two indicators: root mean square error (RMSE) and mean absolute error (MAE), which are conventional indicators to quantify the rating prediction error. The evaluation indexes are shown in equations (20) and (21):

(22) $$\begin{array}{c} \text{RMSE}=\sqrt{\frac{1}{n}\sum_{i=1}^{n}{\left(R_{i}-{\hat{R}}_{i}\right)}^{2}},\\ \text{MAE}=\frac{1}{n}\sum_{i=1}^{n}\left|R_{i}-{\hat{R}}_{i}\right|, \end{array}$$

where *n* is the number of ratings in the dataset, $\hat{R}$ is the predicted score, and *R* is the real score. The smaller the RMSE and MAE, the higher the prediction accuracy.

### 4.3. Baselines

Context-aware recommendation system models can be divided into two types according to feature interaction methods: (A) low-order feature interactions models, which model second-order interactions; (B) deep learning based high-order feature interactions models, which can learn the complex nonlinear relationship between different features.

We choose the following two representative methods of feature interaction types to compare with CA-GNN.

- MF [6], which was proposed in 2009. This is a standard matrix factorization method that represents users and items by latent vectors inferred from observed ratings.
- CAMF-C [15], which was proposed in 2011. CAMF extends MF by considering the impact of context information on project deviations.
- FM [17], which was proposed in 2011. By specifying only the input data, the factorization machine can be easily adapted to various contexts.
- COT [4], which was proposed in 2015. This method uses the context operation matrix generated by the context operation tensor and the latent context vector to model the semantic operation of the user to the context.
- AFM [22], which was proposed in 2017. AFM expands FM by using the attention mechanism to learn the importance of each feature interaction.
- NFM [18], which was proposed in 2017. This is a neural network model for sparse data prediction. Under the framework of the neural networks, the FM is deeply processed to learn the interaction of high-order features.
- AIN [7], which was proposed in 2018. The model can capture the interaction between the context and the user (or item) and get the effect of each context on the user (or item).

### 4.4. Experimental Environment Settings

In the experiment, the dataset is randomly divided into training set (80% sample), verification set (10% sample), and test set (10% sample). The Adam optimizer is used in the training process. GRU network has four feedforward layers, and each layer has 100 hidden units. All experiments were completed in the following environment: Python3.6.2, TensorFlow1.5.0. In the model parameters, learning rate = 0.01, batch size = 512, and epoch = 10.

### 4.5. Experimental Results and Model Comparison

Table 2 is a summary of the performance of different methods, from which we can get the following observations.

1. CAMF has better performance than MF, which proves the effectiveness of context feature information to the recommendation system and highlights the importance of context in the recommendation system.
2. AFM has better performance than FM, which proves the effectiveness of attention under different interactions.
3. Most of the high-order interactive modeling methods are better than the second-order interactive modeling methods. This indicates that the second-order feature interaction is insufficient.
4. We observe that CA-GNN outperforms COT on both datasets, because COT only models the linear high-order feature interaction, and CA-GNN can model the nonlinear high-order feature interaction, which shows that the nonlinear way can better reflect the complex influence relationship between features.
5. The performance of CA-GNN model is better than other methods on Food and Yelp datasets. In the evaluation index RMSE of Food dataset, CA-GNN increased by 2.2% compared with the previous more advanced AIN; in the evaluation index MAE of Food dataset, CA-GNN increased by 1.8% compared with the previous more advanced AIN. In addition, in the evaluation index RMSE of Yelp dataset, CA-GNN increased by 2.3% compared with the previous more advanced AIN; in MAE of Yelp dataset, CA-GNN increased by 2.1% compared with the previous more advanced AIN. This is because of the strong representativeness of graph structure in CA-GNN model and the effectiveness of GNN in node interaction modeling, which shows great advantages over the latest technologies such as AIN.

## 5. Case Analysis

### 5.1. Impact of Context on Users and Items

**Figure 5:**
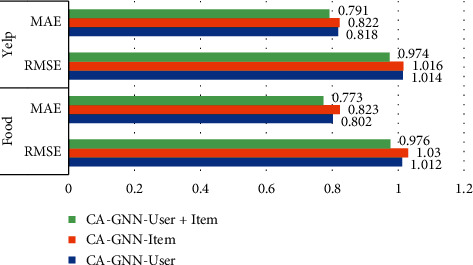
Performance of CA-GNN and CA-GNN variants.

The input data of this paper includes context-user dataset and context-item dataset. To study the impact of context on users and items, we compared the performance of CA-GNN and two variants of CA-GNN. When we only model context-user interactions to get prediction results, CA-GNN-User represents CA-GNN that only models context-user interaction. We can find the influence of context on user behavior. When we only model context-item interactions to get prediction results, CA-GNN-Item represents CA-GNN that only models context-item interactions. We can find the influence of context on the interaction of items. The prediction results of two groups of datasets are shown in Figure 5. In Figure 5, CA-GNN-User refers to CA-GNN that models only user-context interactions, and CA-GNN-Item refers to CA-GNN that models only context-item interactions. CA-GNN-User + Item models both context-user interactions and context-item interactions.

As shown in Figure 5, on the Food and Yelp datasets, CA-GNN-User is slightly better than CA-GNN-Item. This shows that considering context-user interaction can improve recommendation performance more than considering item-context interaction. This may be because user behavior is more affected by the context than project interaction, which reflects user preferences and interests, and user behavior is more helpful to the accuracy of recommendation. Secondly, CA-GNN is superior to CA-GNN-User and CA-GNN-Item in Food and Yelp datasets. This is reasonable because CA-GNN fully considers the impact of context-user interaction and context-item interactions on user and item attributes. Therefore, by considering the impact of context on users and items, performance can be further improved.

### 5.2. Impact of Attention Mechanism

In the calculation of edge weight, we use the attention mechanism. To illustrate the effectiveness of the attention mechanism in CA-GNN, we compared the performance of two versions of CA-GNN with and without attention mechanism. For CA-GNN without attention mechanism, we set all edge weights to 1 (indicating that each node is connected to each other). In Table 3, NoAtt indicates that there is no attention mechanism, and Att indicates that there is an attention mechanism.

As shown in Table 3, CA-GNN with attention mechanism has been considerably improved compared with CA-GNN without attention mechanism. From this result, it can be seen that calculating the edge weight between nodes can improve the accuracy of prediction score.

### 5.3. Impact of Different Contexts and Context Combinations

**Figure 6:**
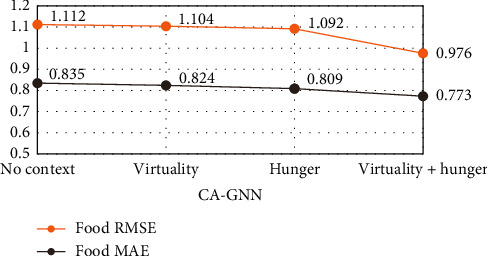
The performance of CA-GNN with different contexts and context combinations on the Food dataset.

**Figure 7:**
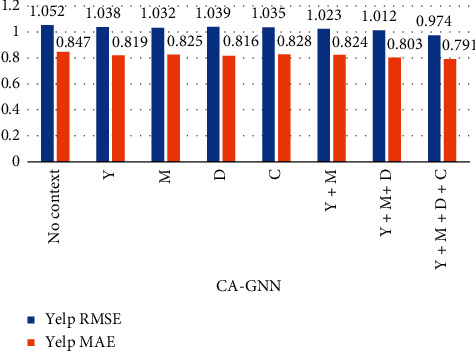
The performance of CA-GNN with different contexts and context combinations on the Yelp dataset.

To evaluate the impact of each context feature and context feature combination on the recommendation results, we further experimented with Food and Yelp datasets containing different context features and context combinations, as shown in Figures 6 and 7. In Figure 7, Y represents year, M represents month, D represent day of the week, and C represents city.

As shown in Figures 6 and 7, when we do not add any context, the effect of score prediction is poor. When only considering the features of a single context feature, the score prediction results are improved, but still not good. When we consider more contextual features, the score prediction results can be further improved. This clearly shows that adding more reasonable context features will help to improve the accuracy of score prediction.

### 5.4. Impact of Degree of Physical Fatigue Features

In the Food dataset, we take the degree of physical fatigue as a contextual feature. An article in Quality Exploration [38] found that when you are too tired and hungry, your body loses too much heat energy and is relatively weak. Eat some sweets, in which sugar can be absorbed by the blood faster than normal foods and quickly replenish your energy. This also shows that the amount of sugar ingested by the body can also reflect the degree of physical fatigue. Therefore, the degree of physical fatigue in the Food dataset is obtained based on the sweetness of the food purchased by the user. There are five levels of food sweetness, namely, very sweet, sweet, medium sweet, slightly sweet, and not sweet. Accordingly, we regard it as five different levels of physical fatigue that are very tired to not tired.

**Figure 8:**
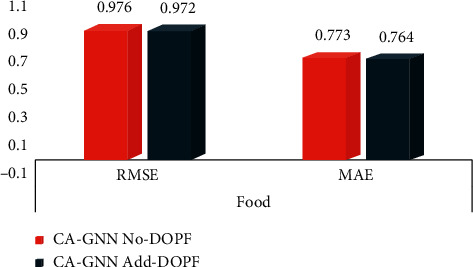
Impact of degree of physical fatigue features.

In Figure 8, No-DOPF means no degree of physical fatigue feature is added, and Add-DOPF means added degree of physical fatigue. As shown in Figure 8, the experimental results of the CA-GNN model show that, in the Food dataset, adding the degree of physical fatigue feature is better than not adding this feature, indicating that considering the degree of physical fatigue feature can help to improve the efficiency of recommendation, and the degree of physical fatigue feature has a positive influence on the prediction result.

## 6. Conclusions and Future Work

In this paper, we point out the shortcomings of previous context-aware recommendation models, including the inability to establish context-user and context-item interactions, the inability to establish context-user/item high-order interactions, and the use of multi-layer perceptrons modeling interaction relationships which will inevitably limit the ability to model complex interactions between different fields in a sufficiently flexible and explicit way. These shortcomings will lead to the model not accurately getting the results of the interaction between the context environment and the user/item and not getting the accurate user behavior prediction results, so it cannot effectively recommend and cannot get the accurate user behavior prediction results. To overcome these limitations, we first proposed representing multi-field features in a graph structure, where each node corresponds to a feature field, and different fields can interact through edges. Therefore, the task of modeling complex context-user interaction and the complex context-item interaction can be transformed into modeling node interactions on feature graphs. This paper designs a new model, CA-GNN, which can model complex interactions between feature fields in a flexible and explicit way. Experiments show that CA-GNN has very good recommendation efficiency on Food and Yelp datasets, especially on the Yelp dataset, which is better than the existing context-aware recommendation system model. Also, the traditional context-aware recommendation system does not use the degree of physical fatigue feature, so the user's needs cannot be accurately judged. Hence, the recommendation result is not ideal. In the experiments of this paper, we used the degree of physical fatigue feature. The experimental results show that the degree of physical fatigue features can help improve recommendation efficiency.

The current context-aware recommendation systems are mostly considering how to integrate context features into the model and establish the interaction between context and user/item. However, few people are studying the quality of context. The same context information may represent different meanings in different contexts or scenes, which leads to the uncertainty of context. Therefore, by improving the quality of context, the accuracy of the recommendation system will be greatly improved. In future work, we will continue to study how to improve the context quality to improve the accuracy of our recommendation model.

## Figures and Tables

**Table 1:** Information statistics of Food and Yelp datasets.

Dataset	Users	Items	Contexts	Interactions	Scale	Field	Features

Food	212	20	2	6360	1–5	4	237
Yelp	96143	49482	4	8021102	1–5	6	191329

**Table 2:** CA-GNN and baseline performance comparison on Food and Yelp datasets.

Model type	Model	Food		Yelp	
		RMSE	MAE	RMSE	MAE

Low-order	MF	1.167	0.950	1.118	0.879
	CAMF-C	1.121	0.900	1.110	0.869
	FM	1.065	0.882	1.099	0.862
	AFM	1.051	0.839	1.092	0.843

High-order	COT	1.055	0.828	1.004	0.835
	NFM	1.013	0.806	1.003	0.824
	AIN	0.998	0.791	0.997	0.812
	CA-GNN	0.976	0.773	0.974	0.791

**Table 3:** CA-GNN performance on Food and Yelp datasets with and without attention mechanism.

Model	Method type	Food		Yelp	
		RMSE	MAE	RMSE	MAE

CA-GNN	NoAtt	1.084	0.797	1.003	0.798
	Att	0.976	0.773	0.974	0.791

## Data Availability

In this paper, we used two datasets, Food dataset and Yelp dataset. The Food dataset was collected by Ono et al. and published in the meeting report (Context-Aware Preference Model Based on a Study of Difference between Real and Supposed Situation Data). The Yelp dataset can be obtained from the website https://www.kaggle.com/yelp-dataset/yelp-dataset.

## References

[1] Adomavicis G., Sankaranarayanan R., Sen S. Context-Awar recommender systems.

[2] Shi Y., Karatzoglou A., Baltrunas L., Larson M., Hanjalic A., Oliver N. Tfmap: optimizing map for top-n context-aware recommendation.

[3] Shi Y., Karatzoglou A., Baltrunas L., Larson M., Hanjalic A. Cars2: learning context-aware representations for context-aware recommendations.

[4] Liu Q., Wu S., Wang L. Cot: contextual operating tensor for context-aware recommender systems.

[5] Wu S., Liu Q., Wang L., Tan T. (2016). Contextual operation for recommender systems. *IEEE Transactions on Knowledge and Data Engineering*.

[6] Koren Y., Bell R., Volinsky C. (2009). Matrix factorization techniques for recommender systems. *IEEE Computer*.

[7] Mei L., Ren P., Chen Z., Nie L., Ma J., Nie J. Y. An attentive interaction network for context-aware recommendations.

[8] Xia Z. Q., Wang Z. T. (2019). Entropy driven collaborative filtering algorithm integrating temporal context. *Journal of Chongqing University of Science and Technology (Natural Science Edition)*.

[9] Meng X. W., Liang B., Du Y. L., Zhang Y. L. (2019). Research on location-based mobile recommendation system utility evaluation. *Journal of Computer Science*.

[10] Adomavicius G., Tuzhilin A. (2011). Context-aware mender systems. *Recommender Systems Handbook*.

[11] Baltrunas L., Amatriain X. Towards time-dependant recommendation based on implicit feedback.

[12] Pannicllo U., Tuzhilin A., Gorgoglione M., Palmisano C., Pedone A. Experimental comparison of pre- vs. postfiltering approaches in context-aware recommender systems.

[13] Peng H. W., Jin Y., LV X. Q., Wang X. L. (2019). A context aware POI recommendation algorithm based on matrix decomposition. *Journal of Computer Science*.

[14] Jin H. (2019). Research on personalized recommendation method of situational awareness based on matrix decomposition. *Science and Technology Trend*.

[15] Baltrunas L., Ludwig B., Ricci F. Matrix factorization techniques for context aware recommendation.

[16] Nguyen T. V., Karatzoglou A., Baltrunas L. Gaussian process factorization machines for context-aware recommendations.

[17] Rendle S., Gantner Z., Freudenthaler C., Schmidt-Thieme L. Fast context aware recommendations with factorization machines.

[18] He X., Chua T.-S. Neural factorization machines for sparse predictive analytics.

[19] Socher R., Chen D., Manning C. D., Ng A. Y. Reasoning with neural tensor networks for knowledge base completion.

[20] Chen J. Y., Zhang H. W., He X. N., Nie L. Q., Liu W., Chua T. S. Attentive collaborative filtering: multimedia recommendation with item- and component-level attention.

[21] Steffen R. (2012). Factorization machines with libFM. *Acm Transactions on Intelligent Systems & Technology*.

[22] Xiao J., Ye H., He X., Zhang H., Wu F., Chua T. S. Attentional factorization machines: learning the weight of feature interactions via attention networks.

[23] Scarselli F., Gori M., Ah Chung Tsoi A. C., Hagenbuchner M., Monfardini G. (2009). The graph neural network model. *IEEE Transactions on Neural Networks*.

[24] Wu S., Tang Y. Y., Zhu Y. Q., Xie X., Tan T. N. Session based recommendation with graph neural networks.

[25] Li Y. J., Tarlow D., Brockschmidt M., Zemel R. (2015). Gated graph sequence neural networks. *Computer Science*.

[26] Li Z. K., Cui Z. Y., Wu S., Zhang X. Y., Wang L. Fi-GNN: modeling feature interactions via graph neural networks for CTR prediction.

[27] Wang Z., Li Y. J., Yan Q., Wang H. W. (2010). A study on the comprehensive evaluation method of mental fatigue in monitoring operation. *Aerospace Medicine and Medical Engineering*.

[28] Ma P., Gao Q. (2020). EEG signal and feature interaction modeling-based eye behavior prediction research. *Computational and Mathematical Methods in Medicine*.

[29] Hochreiter S., Schmidhuber J. (1997). Long short-term memory. *Neural Computation*.

[30] Zeng Y. W., Feng Z., Zhu Y. B., Li Q. (2017). A study on the correlation between blinking times and fatigue based on EEG experiment. *Journal of Changchun University of Science and Technology (Natural Science Edition)*.

[31] Guo H. F., Tang R. M., Ye Y. M., Li Z. G., He X. Q. DeepFM: a factorization-machine based neural network for CTR prediction.

[32] Cho K., Van Merrienboer B., Gulcehre C. (2014). Learning phrase representations using RNN encoder-decoder for statistical machine translation. *Computer Science*.

[33] Wang B. Q., Bi S. B., Dong X. S. (2010). Design and implementation of classification mining based on single -layer perceptron artificial neural network. *Computer Technology and Development*.

[34] Shen C. L., Zhang L., Wu L. Q., Wu S. S. (2019). Sentiment classification towards question-answering based on bidirectional attention mechanism. *Computer Science*.

[35] Cui Z. Y., Li Z. K., Wu S., Zhang X. Y., Wang L. Dressing as a whole: outfit compatibility learning based on node-wise graph neural networks.

[36] Song W. P., Shi C., Xiao Z. P. AutoInt: automatic feature interaction learning via self-attentive neural networks.

[37] Ono C., Takishima Y., Motomura Y., Asoh H. Context-aware preference model based on a study of difference between real and supposed situation data.

[38] Anon (2009). When sweet food is good for health. *Quality Exploration*.

